# One-Year Pilot Study Results of Newborn Screening for Spinal Muscular Atrophy in the Republic of Croatia

**DOI:** 10.3390/ijns10030050

**Published:** 2024-07-16

**Authors:** Darija Šimić, Ana Šarić, Ana Škaričić, Ivan Lehman, Branka Bunoza, Ivana Rako, Ksenija Fumić

**Affiliations:** 1 Department of Laboratory Diagnostics, University Hospital Center Zagreb, 10000 Zagreb, Croatia; darija.simic1@kbc-zagreb.hr (D.Š.); ana.saric995@gmail.com (A.Š.); ana.skaricic@kbc-zagreb.hr (A.Š.); ivana.rako@kbc-zagreb.hr (I.R.); 2 Department of Pediatrics, University Hospital Center Zagreb, 10000 Zagreb, Croatia; ivan.lehman@kbc-zagreb.hr (I.L.); brankabunoza@gmail.com (B.B.)

**Keywords:** newborn screening, pilot project, spinal muscular atrophy

## Abstract

Spinal muscular atrophy (SMA) is a neuromuscular and neurodegenerative disease caused by the homozygous deletion of *SMN1* exon 7 in 95% of cases. The prognosis for SMA patients has improved with the development of disease-modifying therapies, all of which are available in Croatia. The best treatment outcomes occur when therapy is applied before symptoms appear, making newborn screening (NBS) for SMA a crucial factor. Since SMA NBS is the first genetic test performed in our laboratory, for successful implementation of the program, we had to overcome logistical and organizational issues. Herein, we present the results of the SMA NBS during the one-year pilot project in Croatia and verify the suitability of the Targeted qPCR^™^ SMA assay for SMA NBS. The pilot project started on 1 March 2023 in the Department for Laboratory Diagnostics of the University Hospital Center Zagreb. A total of 32,655 newborns were tested. Five SMA patients were detected, and their diagnoses were confirmed by the multiplex ligation-dependent probe amplification (MLPA) assay. There have been no false positive or false negative results, to our knowledge so far. The incidence of SMA determined during the pilot study is consistent with the SMA incidence data from other European countries.

## 1. Introduction

Spinal muscular atrophy (SMA) is a neurodegenerative and neuromuscular disease inherited in an autosomal recessive manner, with the estimated incidence in Europe at about 1:10,000–1:5000 live births [1]. In about 95% of cases, SMA is caused by the homozygous deletion of exons 7 and 8, or just exon 7 of the survival motor neuron (*SMN1*) gene in the 5q13 locus [2], which encodes the SMN protein. The absence of the SMN protein triggers the degeneration of anterior horn cells within the spinal cord, leading to symmetrical muscle weakness and atrophy [2].

Besides the telomeric *SMN1*, chromosome 5 also carries the centromeric *SMN2* gene, which originated as a result of *SMN1* duplication. In SMA patients, the *SMN2* copy number is a positive modifier of SMA phenotype, as a higher *SMN2* copy number usually results in milder clinical manifestations with later disease onset [3]. The clinical picture of SMA is usually divided into five subtypes, depending on the age of symptom onset, the degree of motor development [4], and the highest motor function achieved.

Until recently, SMA was one of the most common genetic causes of death in early infancy, but with the new treatment options currently available, the death rate has decreased greatly. Many new diagnostic tests that enable early detection of the disease have also been developed worldwide, and it is recommended that newborn screening (NBS) for SMA is introduced in all European countries by 2025 [5]. Herein, we report the results of a one-year SMA NBS pilot project in the Republic of Croatia, which aimed to introduce SMA screening into the daily routine work of the NBS laboratory. Implementing a new target disease into the NBS laboratory can impact the existing analytical procedures in terms of economic aspects, new technology, sample distribution, and personnel education [6]. Our laboratory was confronted with all these demands, which will be described in more detail in this paper.

## 2. Materials and Methods

The SMA NBS pilot project started on 1 March 2023 and ended on 1 March 2024. We opted for real-time polymerase chain reaction (real-time PCR) analysis for the first-tier SMA screening test using Targeted qPCR^™^ SMA reagent kit (LaCAR MDx Technologies/ZenTech, Liège, Belgium). This test detects the homozygous deletion of exon 7 of the *SMN1* gene. It is based on DNA extraction from a dried blood spot (DBS), followed by qPCR. The DBS samples of neonates are collected between 48 and 72 h of life on a standardized Whatman^™^ 903 filter paper card, which is also used for all other NBS tests in our laboratory. A total of 32,655 newborns were tested. The reagent kit was internally verified following the guidelines of our Quality Control Department. For this purpose, a substantial cohort comprising both SMA positive and negative samples, previously analyzed by the multiplex ligation-dependent probe amplification (MLPA) assay, underwent the analysis using the kit.

In the first step of sample preparation for SMA screening, a single 3.2 mm DBS punch was treated with Lysis Solution^®^ and heated to 95 °C for 30 min for lysis of the blood cells. In the following step, an Extraction Buffer^®^ was added to extract the DNA from white blood cells. The qPCR mix was prepared in a separate room from the DNA extraction step under sterile conditions. Then, 2 µL of isolated DNA was loaded in a qPCR plate pre-filled with a commercial mixture (MasterMix). A qPCR analysis was performed on the Bio-Rad CFX96^™^ Dx system (Bio-Rad, Hercules, California, USA). MasterMix contains highly specific fluorescent probes complementary to *SMN1* and *RPP30*. A probe exclusively targeting exon 7 of *SMN1* was labeled with FAM fluorophore, while the probe targeting *RPP30* was labeled with HEX fluorophore. *RPP30* is an endogenous control whose expression level should not differ significantly between samples. The total procedure for the sample preparation and results analysis lasted approximately three hours.

In the case of a positive SMA screening, the analysis was urgently repeated from the same DBS sample in triplicate. If a child tested positive from repeated analysis, an MLPA assay (P021-B1 SMA, MRC-Holland, Amsterdam, The Netherlands) was performed from a fresh EDTA-blood sample to confirm the homozygous deletion of *SMN1* exon 7 and to determine the number of copies of *SMN2* and *NAIP*.

## 3. Results

**Figure 1:**
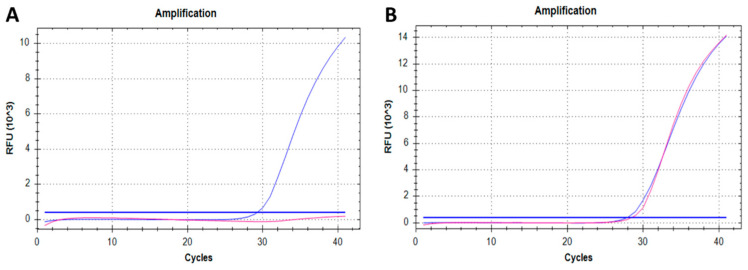
First-tier test (qPCR) results for SMA NBS: amplification curves for (**A**) positive SMA screening and (**B**) negative SMA screening. The pink curves represent the fluorescent signal of FAM fluorophore (*SMN1*), whereas the blue curves represent the fluorescent signal of HEX fluorophore (endogenous control *RPP30*).

The amplification curves for *SMN1* and *RPP30* showed a characteristically similar sigmoidal shape and reached the exponential phase in healthy individuals (Figure 1B). Only HEX fluorescent signal representing *RPP30* was observed for SMA patients’ samples, while an FAM fluorescent signal representing *SMN1* was absent (Figure 1A).

The quantification cycle (C_q_) values for healthy infants ranged between 28 and 32 for both *SMN1* and *RPP30*. The mean *SMN1* C_q_ value for healthy infants was 29.16. In SMA patients, C_q_ for *RPP30* also ranged between 28 and 32, whereas C_q_ for *SMN1* was greater than 35 or undetected.

**Figure 2:**
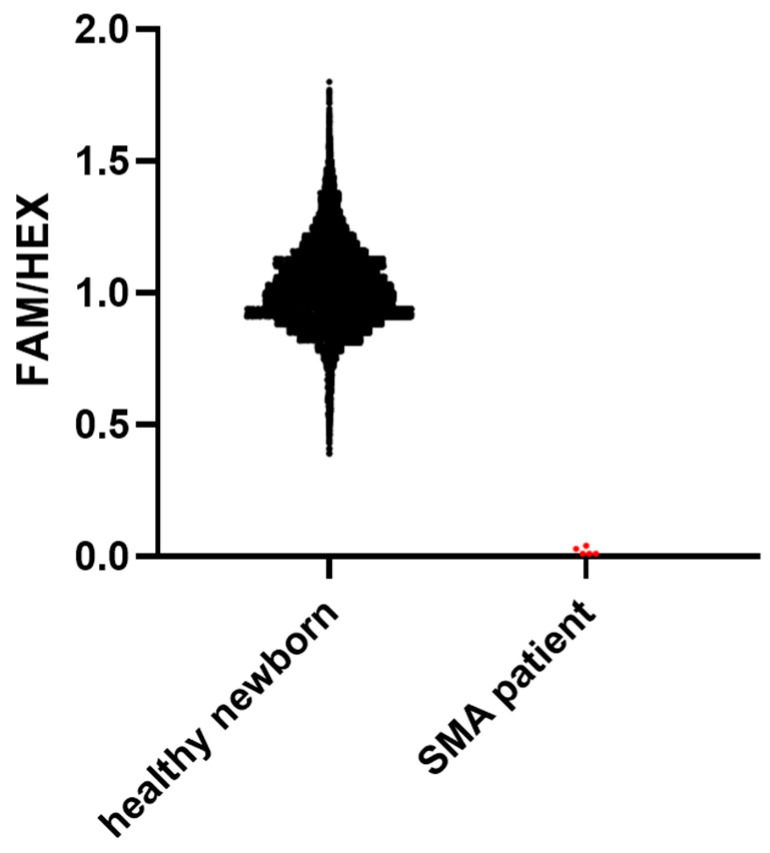
Dot plot representation of the ratios of FAM to HEX end-point fluorescence for healthy newborns and SMA patients. Values of 32,650 healthy newborns and 5 SMA patients are represented with black and red dots, respectively (GraphPad Prism 8.0.1).

The results are displayed as the ratio of the final fluorescence value obtained for FAM to the final fluorescence value obtained for HEX signal (end-point FAM/HEX) from the same sample. A ratio lower than 0.26 was considered a positive SMA screening result. The end-point FAM/HEX ratios ranged from 0.39 to 1.9 for healthy newborns, with a mean value of 1.026. The ratios for SMA patients ranged from 0.01 to 0.04, with a mean value of 0.03 (Figure 2). When the initial results fell within the range of 0.26 to 0.4, an analysis was repeated as a precautionary measure. A few samples that exhibited low end-point FAM/HEX ratios but were still in the “healthy” range were tested with an MLPA assay, all revealing heterozygous genotypes. Interpretation of the runs was also confirmed using the interpretation software GeneFoxCub 1.0.0-RUO provided by LaCAR MDx Technologies (Liège, Belgium) along with the kit.

During the one-year pilot study, five newborns screened positive, and the SMA diagnoses were confirmed in all five cases with MLPA assays, indicating an incidence of 1:8163. There were no false positive results, as all the positive screening results were confirmed by the MLPA. There were also no false negative results, to our knowledge so far, as no SMA has been clinically diagnosed outside the screening cohort. For all DBS samples received by the laboratory, the median age for SMA NBS results reported was 6 days (range 2–74). The median age for the diagnosis of SMA was 11 days (range 11–13), and the median age for therapy application was 31.5 days (range 25–41).

## 4. Discussion

In the not-too-distant past, SMA used to be one of the most common genetic diseases contributing to infant mortality [7]. Type 1 SMA, which accounts for 60% of cases, appears in early infancy and has a severe clinical course; untreated, it results in death or the need for permanent ventilation before the age of two [4]. The severity of SMA is largely determined by the number of *SMN2* copies, where two copies typically result in the most severe form, while four or more copies lead to a milder form of the disease [4]. *SMN1* and *SMN2* are paralogous genes as their sequences differ only in 20 nucleotides [8] and they code for the same amino acid sequence. However, mutation c.840C>T in exon 7 of *SMN2* leads to exon skipping in the majority of *SMN2* pre-mRNA transcripts, resulting in a truncated and nonfunctional protein, which is rapidly degraded after the translation (SMN-∆7) [3]. As this region of chromosome 5 is prone to rearrangements, the number of *SMN2* copies is highly variable between individuals, ranging from 0 to 8 [9].

With the approval of disease-modifying therapies (DMTs) that alter the natural course of SMA, the prognosis for SMA patients has significantly improved [10]. Two of the current therapeutic approaches, nusinersen and risdiplam, target *SMN2* translation to produce a normal, full-length SMN protein from *SMN2* mRNA. The third therapeutic approach, onasemnogene abeparvovec, is a gene replacement therapy that delivers *SMN1* via the adeno-associated viral vector (AAV9) [11].

Because of rapid motor neuron degeneration, which can start as early as embryonal development in type 0 SMA [12], the best results of treatment are achieved if the therapy is applied early in the disease course or before the symptoms of the disease appear [13]. This led to the rapid development and implementation of SMA NBS programs globally [14]. The purpose of NBS programs is to achieve a timely presymptomatic diagnosis of treatable disorders, which allows for early initiation of therapy and thus a reduction in morbidity and mortality [15]. It is estimated that SMA NBS is currently available for about 58% of newborns in geographical Europe or for about 65% of newborns including surrounding countries [16].

The Croatian NBS laboratory, located in The Department of Laboratory Diagnostics of Hereditary Metabolic Diseases and Newborn Screening in the University Hospital Center Zagreb, has been screening for phenylketonuria and congenital hypothyroidism since 1978 and 1985, respectively. In 2017, six additional diseases were added to the NBS program: isovaleric aciduria, glutaric aciduria type I, carnitine uptake deficiency, medium-chain acyl-CoA-dehydrogenase deficiency, very long-chain acyl-CoA-dehydrogenase deficiency, and long-chain 3-OH-acyl-CoA-dehydrogenase deficiency (isolated or as part of a trifunctional protein deficiency). To ensure the quality of the obtained screening results, our laboratory is accredited following the norm HRN EN ISO 15189:2012 [17] and we participate in the CDC’s Newborn Screening Quality Assurance Program. The laboratory operates from Monday to Saturday in two shifts.

In 2023, the pilot project for SMA NBS was implemented into the existing screening panel. For the successful start of the program, it was essential to coordinate a series of procedures with the support of all participants, including health professionals and parents/guardians. The deadline for implementation was significantly shortened due to public pressure, and we faced numerous challenges. A helpful starting point was the two-day training dedicated to SMA NBS- “Before SMA: The Academy”, organized by Prof. Laurent Servais and Dr. Tamara Dangouloff. The academy is held annually in Liège, Belgium, and is an excellent opportunity for networking and learning about SMA NBS from experienced child neurologists and geneticists.

The first step was the approval of the project proposal by the Ministry of Health, accompanied by ensuring financial resources. The costs of SMA NBS, including training personnel, purchasing reagents, and new equipment, were incorporated into the pilot project budget. In smaller countries like Croatia, there is often no representative office for procuring suitable reagents and equipment, particularly for specialized areas such as newborn screening. The hospital tender had to be adjusted to include a new type of reagent and the additional laboratory consumables used. Moreover, before the introduction of SMA screening, the NBS laboratory only performed biochemical analyses. Therefore, providing a sterile space for handling DNA samples was necessary. The latter was made possible thanks to a pediatrician who gave up his office located next to the laboratory, which was then remodeled and equipped with a UV lamp, a UV sterilization cabinet, and two qPCR devices. The NBS laboratory staff had no previous experience in molecular techniques, so two molecular biologists were employed to perform the analyses and educate colleagues about working with DNA specimens. Alongside molecular biologists, two neuropediatricians from the Reference Center of the Ministry of Health of the Republic of Croatia for Childhood Neuromuscular Diseases and Clinical Electromyoneurography were recruited. The team has weekly meetings and communicates the screening results daily in a dedicated WhatsApp group.

**Figure 3:**
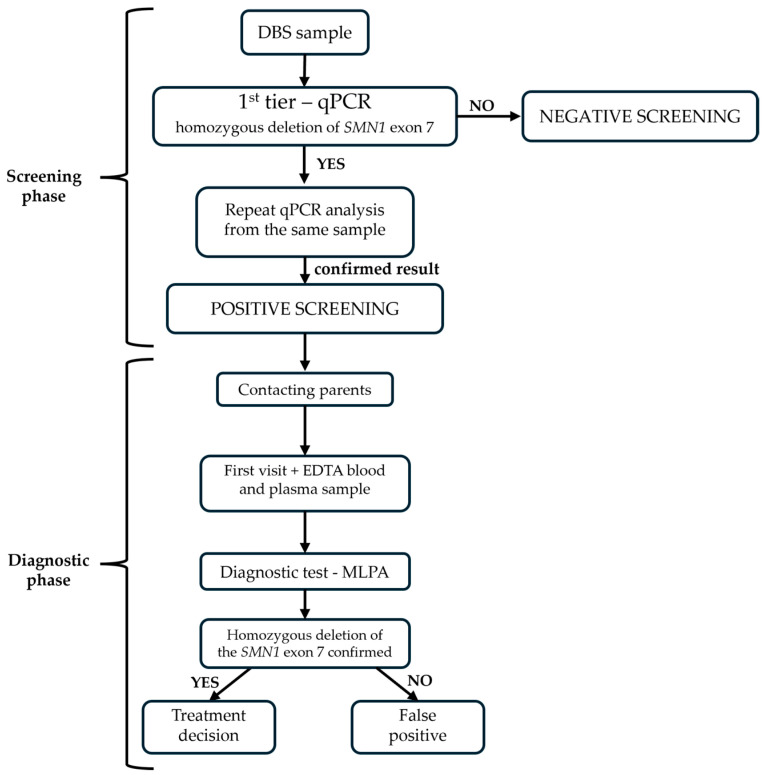
Workflow in SMA NBS.

Medical staff of all maternity hospitals in Croatia were informed about the expansion of NBS and the importance of proper DBS sampling, such as avoiding all potential sources of contamination and qPCR reaction inhibitors (blood dripping from a container with anticoagulant EDTA and heparin) [18,19]. Public information about NBS, available at the official website of the University Hospital Center Zagreb, was updated [20]. Lastly, the SMA NBS workflow was established and adapted to the existing working conditions in the laboratory (Figure 3).

As our laboratory is the only NBS laboratory in Croatia, we receive and analyze 150–200 neonates’ DBS samples daily from all maternity hospitals, as well as from children born at home. Each DBS sample card contains information about the mother and the infant, and instructions on proper sampling of the blood on the back of the card. NBS is mandatory for newborns in Croatia, so individual agreement to NBS is not required; however, parents have the right to refuse NBS and, in such cases, are obliged to sign a statement of refusal. During the SMA NBS pilot study, 0.03% of parents/guardians declined screening, with the majority of refusals occurring early in the study. As the implementation was widely covered in the media, there was an inevitable spread of negative and inaccurate comments about SMA NBS through social networks. This likely caused fear among pregnant women and consequently led to an increased number of screening refusals.

In SMA NBS, a positive screening result is immediately communicated by the laboratory to the neuropediatricians. The neonate’s parents or guardians are contacted the same day by the neuropediatrician and urgently invited in for an examination. During the first examination of the newborn, parents are provided with information regarding the disease and therapeutic options. With parental consent, a venous blood sample of the neonate is taken for a confirmatory MLPA assay, also performed in our department, and a plasma sample is sent to a collaborative laboratory for AAV antibody titer measurement. MLPA is a semi quantitative assay for the detection of copy number variants (deletions or duplications) in *SMN1*, *SMN2,* and exon 5 of the *NAIP* genes in genomic DNA. Although *SMN2* copy number is the main factor in estimating the severity of the clinical presentation of SMA and essential in the decision on the choice of therapy, it is important to note that the clinical presentation may be influenced by other modifying factors besides the *SMN2* copy number [14].

Through the Croatian SMA NBS pilot project, we have detected five SMA patients, with the calculated incidence of 1: 8163 being similar to other European countries [21]. The *SMN1*-to-*RPP30* ratios displayed a Gaussian distribution; therefore, no conclusion about the copy number of *SMN1* of the samples can be made. Out of the five SMA patients, one had two *SMN2* copies, two had three *SMN2* copies, one had four *SMN2* copies, and one had six *SMN2* copies (Table 1). No newborns had signs of SMA at the time of their first referral to the neuromuscular reference center. Parents of two newborns opted for risdiplam, one opted for onasemnogene abeparvovec, and one opted for nusinersen. One SMA patient has not received any treatment because of six *SMN2* copies (Case 2) but is under constant supervision and, so far, shows no clinical signs of the disease. One of the detected SMA patients is a resident of Bosnia and Herzegovina (Case 3) but entered the screening cohort because she was born in a Croatian maternity hospital. This patient is currently being treated and followed in Bosnia and Herzegovina and is not included in the calculated incidence.

In addition to the program’s unquestionable advantages, we must also be aware of its limitations, with the most prominent being the small number of detected patients and the relatively short period of their follow-up. The number of cases per individual countries is low, and it is essential to bring together diagnostic and treatment data to draw relevant conclusions. A certain number of patients detected by screening will already be symptomatic at the time of diagnosis due to a broad phenotypic spectrum of the disease. In about 5% of cases, other mutations of distinct exons and introns in *SMN1* induce an SMA clinical phenotype [3], and these cases cannot be detected by currently available NBS tests. Moreover, in the era of more than one available DMT for SMA, NBS raises ethical questions [1]. One of them is the financial burden due to the high cost of DMTs. Certainly, the total financial and hospitalization costs (including DMT) are somewhat lower for treated patients identified by NBS, compared to patients treated after they develop symptoms [4,22]. However, the availability of DMTs varies between countries, with each country having different guidelines for the application of therapies. This leads to unequal choices for optimal treatment in patients detected both symptomatically and through screening programs. In Croatia, access to treatment for SMA patients has improved since the implementation of the SMA NBS program, with the most significant change being that gene therapy has become available to patients with two and three *SMN2* copies. The current Croatian guidelines for SMA DMTs are shown in Table 2 [23].

The psychosocial impact of NBS on families is still uncertain, possibly due to the lack of data on the long-term effects of the treatment and/or a “wait-and-see” strategy in SMA patients with >4 *SMN2* copies [15] which is applied in our country. A study from Germany revealed that families with patients diagnosed through SMA NBS are greatly affected by the burden of the disease in various aspects of their lives, even after timely treatment and improved neurological outcomes [15]. Parents/caregivers, who come from various backgrounds and possess different levels of education, have to make a decision about the choice of therapy in a very short period of time. For this reason, it is of crucial importance to have a multidisciplinary counseling team at their disposal. Strong psychological support is not only important in the first days after the diagnosis but also in the further stages of accepting their child’s chronic illness [15].

## 5. Conclusions

During the one-year SMA NBS pilot study in Croatia, we have screened 32,655 neonates and identified 5 SMA patients whose diagnoses were confirmed by MLPA in the first two weeks of life. This allowed for an early start to treatment and prevented progression of the disease. All detected infants are under constant supervision, and they are thriving. We did not encounter false positive nor false negative results, to our knowledge, and the performance of the SMA screening test is shown to not be affected by poorly sampled DBS. Moreover, the pilot project did not have any negative impact on the performance of the existing NBS program, which showed that it is possible to add more diseases into the screening panels without disrupting the well-established laboratory workflow.

## Figures and Tables

**Table 1:** Screening and diagnostic timeline (in post-natal days) for SMA patients identified by SMA NBS.

Case ID	Sex	DBS Received in the NBS Laboratory	Screening Results (qPCR)	First Appointment at the Neuromuscular Center	Symptoms at Referral	Official Diagnosis (MLPA Results)	*SMN2* Copy Number	Anti AAV9	Age at Treatment/Treatment Option
1	F	4	5	6	no	12	3	negative	25/onasemnogene abeparvovec
2	M	3	4	patient was still at the maternity hospital	no	11	6	positive	-/follow-up with the patient
3	F	11	11	12	no	13	2	negative	41/nusinersen patient is a resident of Bosnia and Hercegovina
4	F	8	9	10	no	11	3	-	23/risdiplam
5	M	8	8	9	no	11	4	negative	38/risdiplam

**Table 2:** Access to treatment for SMA patients in Croatia.

Therapy	Guidelines
nusinersen	≥2 *SMN2* copies patients with SMA types 1, 2, or 3
risdiplam	1–4 *SMN2* copies patients with SMA types 1, 2, or 3
onasemnogene abeparvovec	≤12 months of age with SMA type 1 presymptomatic children with ≤3 *SMN2* copies

## Data Availability

The data are not publicly available due to privacy restrictions.
